# EEG Markers of Treatment Resistance in Idiopathic Generalized Epilepsy: From Standard EEG Findings to Advanced Signal Analysis

**DOI:** 10.3390/biomedicines10102428

**Published:** 2022-09-28

**Authors:** Emanuele Cerulli Irelli, Giorgio Leodori, Alessandra Morano, Carlo Di Bonaventura

**Affiliations:** 1 Department of Human Neurosciences, Sapienza University of Rome, 00185 Rome, Italy; 2 IRCCS Neuromed, 86077 Pozzilli, Italy

**Keywords:** drug resistance, prolonged ambulatory EEG (paEEG), 24-h EEG, resting-state EEG, machine learning, quantitative EEG, juvenile myoclonic epilepsy (JME), childhood absence epilepsy (CAE), graph theory, functional connectivity

## Abstract

Idiopathic generalized epilepsy (IGE) represents a common form of epilepsy in both adult and pediatric epilepsy units. Although IGE has been long considered a relatively benign epilepsy syndrome, a remarkable proportion of patients could be refractory to treatment. While some clinical prognostic factors have been largely validated among IGE patients, the impact of routine electroencephalography (EEG) findings in predicting drug resistance is still controversial and a growing number of authors highlighted the potential importance of capturing the sleep state in this setting. In addition, the development of advanced computational techniques to analyze EEG data has opened new opportunities in the identification of reliable and reproducible biomarkers of drug resistance in IGE patients. In this manuscript, we summarize the EEG findings associated with treatment resistance in IGE by reviewing the results of studies considering standard EEGs, 24-h EEG recordings, and resting-state protocols. We discuss the role of 24-h EEG recordings in assessing seizure recurrence in light of the potential prognostic relevance of generalized fast discharges occurring during sleep. In addition, we highlight new and promising biomarkers as identified by advanced EEG analysis, including hypothesis-driven functional connectivity measures of background activity and data-driven quantitative findings revealed by machine learning approaches. Finally, we thoroughly discuss the methodological limitations observed in existing studies and briefly outline future directions to identify reliable and replicable EEG biomarkers in IGE patients.

## 1. Introduction

Idiopathic generalized epilepsy (IGE) accounts for almost 20% of all epilepsies in adults and children and is associated with a strong genetic background [1]. IGE has been subdivided into four distinct clinical syndromes: childhood absence epilepsy (CAE), juvenile absence epilepsy (JAE), juvenile myoclonic epilepsy (JME), and IGE with generalized tonic-clonic seizures alone [2]. Although some clinical and electroencephalography (EEG) differences can be observed across different syndromes, they present a strong clinical overlap and share genetic causative factors, suggesting a common pathophysiological background [2].

IGE has traditionally been considered to have a favorable prognosis, though increasing evidence has shown that up to 40% of patients are refractory to treatment and a proportion of them may experience unremitting seizures throughout their clinical history [3]. Drug resistance has been consistently associated with an increased risk of mood disorders and poorer quality of life, with important limitations in education, employment opportunities and obtaining a driving license, and with a greater stigma for patients as well [4]. Considering the importance of seizure freedom to obtain optimal clinical and psychosocial outcomes, the early identification of patients who are likely to develop drug resistant epilepsy is critical. However, although some clinical factors have been found to help predict drug resistance in IGE patients, the impact of EEG in this setting is controversial and different studies have come to remarkably different conclusions [5]. The majority of existing literature has been focused on the role of routine EEG, and only recently a renewed interest in the potentialities of sleep EEG and 24-h EEG monitoring has been observed in light of the occurrence of some EEG patterns prominently during the sleep state [6]. In addition, the application of computational techniques to the processing of EEG signals has enabled researchers to investigate and analyze subtle dynamics that are not readily detectable by visual inspection alone, allowing them to explore the potential role of advanced EEG analysis in identifying new biomarkers of treatment resistance in IGE [7].

In this manuscript, we briefly review the EEG features commonly observed in IGE patients and analyze those associated with drug resistance by examining the findings observed during both routine EEG and prolonged ambulatory EEG recordings (paEEG). We also provide an overview of the recent discoveries in the field of advanced EEG analysis. All studies were evaluated with special attention to the methodological limitations. Finally, we discuss future directions to identify reliable and replicable EEG biomarkers in IGE patients.

A literature search for relevant articles was carried out on online databases (EMBASE, MEDLINE) covering a period from 1980 to March 2022 using the following medical subject headings: IGE (idiopathic generalized epilepsy, genetic generalized epilepsy, GGE), EEG (EEG, electroencephalography, resting state, prolonged EEG, 24-h EEG, ambulatory EEG), drug resistance (drug resistance, treatment resistance, treatment response, seizure freedom, pharmacoresistance, medication refractoriness), biomarkers (markers, features, patterns, findings), and prognosis. 

## 2. EEG Features of Idiopathic Generalized Epilepsy

**Figure 1:**
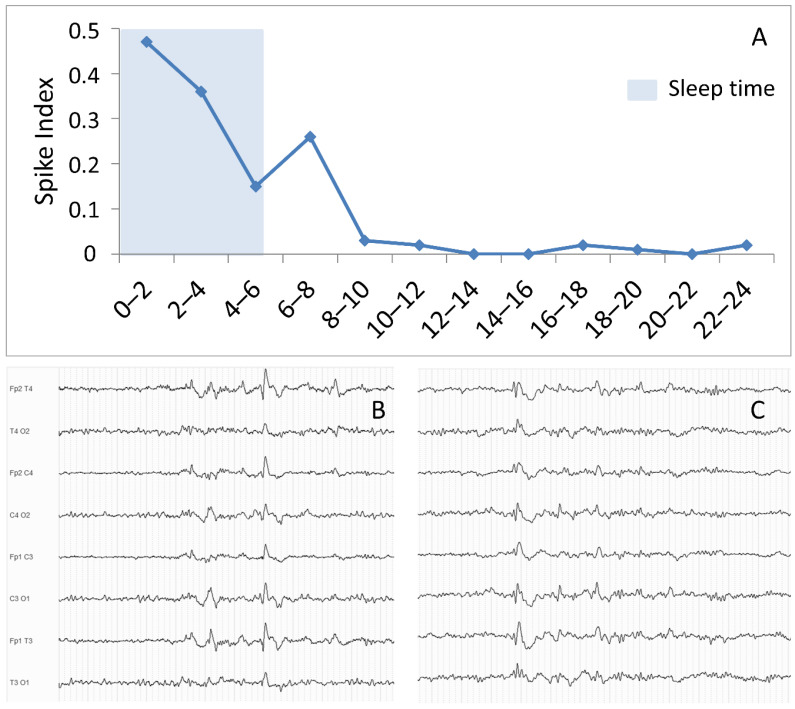
Circadian distribution and morphology of epileptiform discharges. Panel (**A**): Distribution of epileptiform discharges (ED), measured as by spike index, during a 24-h EEG recording in a sample patient with idiopathic generalized epilepsy. The blue shaded area represents the sleep period. Spike index was calculated as (duration of ED -measured in minutes—occurring during a 2-h interval/120 min) × 100. Panel (**B**,**C**): examples of ED in the midst of sleep physiological figures.

The EEG hallmark of IGE syndromes is the occurrence of interictal symmetric, bisynchronous, and regular generalized spike-wave discharges (GSWDs) and generalized polyspike-wave discharges (GPSWDs) in the context of normal EEG background activity. Interictal discharges are usually more frequent during non-rapid eye movement sleep (NREM) and in the first hour after awakening (Figure 1) [7]. The frequency of these generalized discharges usually ranges from 3 to 6 Hz, with higher frequencies possibly occurring in JME patients when compared to other IGE syndromes [7]. During NREM sleep, GSWDs and GPSWDs may exhibit a slower frequency (<3 Hz) and appear intermingled with K-complexes and sleep spindles (Figure 1) [8], whereas during rapid eye movement sleep they usually appear with the same morphology and frequency as during wakefulness. 

While ictal and interictal EEG patterns are almost indistinguishable in absence seizures, different ictal EEG patterns are usually observed in myoclonic and generalized tonic-clonic seizures. The ictal EEG pattern in the latter is typically characterized by a diffuse attenuation in EEG amplitude followed by generalized rhythmic activity that increases in amplitude and decreases in frequency (tonic phase), eventually followed by repetitive polyspike-wave complexes (clonic phase) [9]. In myoclonic seizures, the jerks usually appear concurrently with bursts of generalized polyspikes of 10–16 Hz, usually followed by slow waves. The resulting polyspike-wave pattern may persist for several seconds after the myoclonic jerk terminates [9]. 

Atypical interictal EEG abnormalities can also be frequently encountered, such as focal slowing or focal epileptiform discharges (EDs), asymmetric or asynchronous discharges, irregular morphology of GSWDs/GPSWDs, and generalized fast discharges (namely generalized paroxysmal fast activity, -GPFA-, and generalized polyspike trains, -GPT-, which will be discussed in the paragraph regarding sleep EEG). The reported frequency of these atypical EEG findings is highly variable across different studies (ranging from 13% to 66.4%) due to the heterogeneous criteria used to define them and classify IGE syndromes, as well as the methodological discrepancies among studies (e.g., age at reviewed EEG, use of pre-treatment or post-treatment EEG, etc.) [10,11]. The prognostic significance of these atypical findings will be discussed in the next chapters.

When considering activation procedures, hyperventilation and intermittent photic stimulation are the most widely used during routine EEG recordings. Hyperventilation may facilitate EDs and typically triggers absence seizures in CAE/juvenile absence epilepsy patients [7]. Intermittent photic stimulation may determine a photoparoxysmal response (PPR), defined as the occurrence of ED time-locked and reproducible with intermittent photic stimulation. PPR may be restricted to occipital areas or more generalized, and it may be associated with clinical manifestations. PPR is certainly more common among patients with JME (30%) when compared with other syndromes [12]. Another EEG feature that may occur together with PPR is eye closure sensitivity, which reflects the occurrence of ED appearing within 1–3 s and lasting 1–4 s after eye closure [7]. Eye-closure sensitivity must be distinguished by fixation-off sensitivity, which is an exceptional finding in IGE and is defined as the occurrence of occipital or generalized EDs induced by elimination of central vision and fixation (e.g., by keeping the eye closed during routine EEG, wearing Frenzel or spherical lenses, etc.) [13]. In addition to intermittent photic stimulation-sensitivity, photosensitive IGE patients may also be reactive to visual patterns, typically stripes. Interestingly, a few pattern-sensitive patients have been described as being unresponsive to conventional intermittent photic stimulation [14]. In addition, different kinds of cognitive stimuli can trigger seizures in IGE subjects, ranging from verbal (reading, writing, etc.) to non-verbal cognitive tasks, such as in thinking-induced (e.g., arithmetic activity, etc.) and praxis-induced seizures (Rubrik’s cube, written calculations, playing chess, etc.) [15]. According to some authors, these activation techniques should also be incorporated in routine EEG studies to elicit EDs in IGE patients, with the aim of increasing the diagnostic yield of EEG recordings [15]. 

**Figure 2:**
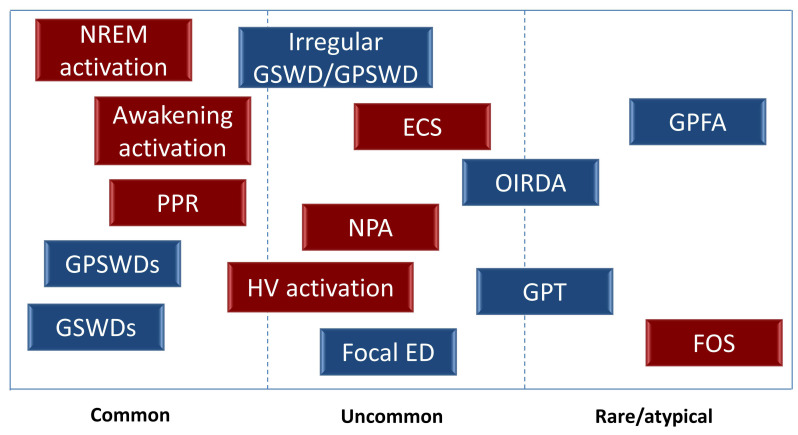
EEG patterns in idiopathic generalized epilepsy. Electroencephalography (EEG) patterns according to the frequency of their occurrence. Spontaneous EEG patterns are indicated in blue, whereas EEG patterns activated by specific conditions/procedure are indicated in red. Abbreviations: ECS = eye closure sensitivity; ED = epileptiform discharges; FOS = fixation-off sensitivity; GPFA = generalized paroxysmal fast activity; GPSWD = generalized polyspike-wave discharge; GPT = generalized polyspike train; GSWD = generalized spike-wave discharge; HV = hyperventilation; NPA = neuropsychological activation; OIRDA: Occipital Intermittent Rhythmic Delta Activity; PPR = photoparoxysmal response.

An overview of the frequency of the above-mentioned EEG patterns is illustrated in Figure 2.

## 3. EEG Features Associated with Treatment Resistance Observed during Routine EEG Recordings

### 3.1. Focal Abnormalities

During the previous decades, several authors have tried to identify possible EEG findings that predict treatment response. Special attention has been paid to the prognostic significance of atypical EEG findings, particularly focal EEG abnormalities, during routine exams. Matsuoka first reported the association between focal EEG abnormalities and poor treatment response in JME patients [16], and his results were then replicated in other studies [17,18,19,20]. However, this association was not confirmed by other authors in various IGE syndromes [21,22,23]. The retrospective design of these studies, the lack of precise EEG criteria to define focal abnormalities, and the use of EEG recorded at different timepoints during follow-up hampered the possibility to compare these results and draw solid conclusions. Such limitations were partially overcome by Japaridze et al., who prospectively investigated the prognostic impact of well-defined focal EEG abnormalities in a cohort of 168 juvenile absence epilepsy and JME patients, 96 of whom were drug naïve [24]. The authors did not find any association between focal EEG abnormalities and seizure outcome at 12 months, even though the low rate of treatment resistance (9.3%) during follow-up could have contributed to a type II alpha error. A combined qualitative and quantitative approach to define focal EEG abnormalities was used by Karakis et al., who retrospectively reviewed the EEGs of 51 drug-naïve IGE patients and confirmed the lack of any association between focal EEG abnormalities at baseline and refractoriness to medical therapy at 6 months [25]. The negative results of these two well-designed studies dampened the interest in focal abnormalities as prognostic biomarkers of drug resistance in IGE, even though the relatively small sample size and short follow-up of both studies did not allow the issue to be definitively resolved. 

### 3.2. Photoparoxysmal Response 

Another debated EEG variable is represented by PPR. The most solid evidence comes from studies evaluating PPR in CAE patients prior to initiating antiseizure medications (ASM). In a retrospective study, Incecik et al. reviewed the EEGs of 52 drug-naïve CAE patients and evaluated the baseline variables predicting failure to the first prescribed ASM [26]. They found that PPR was the only factor that predicted a lack of medication response in multivariable survival analysis. Similarly, Covanis et al. found that the presence of marked photosensitivity predicted relapse after ASM withdrawal in CAE patients [27]. These findings were not replicated by another study performed in CAE patients, though this work was biased by the small representation of drug-resistant CAE (nine patients) and by the lack of systematic use of baseline EEG to study seizure prognosis [28]. In the well-designed Dutch study of epilepsy, none of the 47 CAE patients (selected according to strict criteria) seemed to display PPR, and the baseline EEG was not found to predict the long-term prognosis of these patients [29]. However, the interpretation of the prognostic significance of PPR in CAE is controversial, since some authors identified CAE patients with PPR as a distinct sub-phenotype with an unfavorable outcome, and others even considered PPR as an exclusion criterion for CAE [30,31,32]. As regards IGE in general, PPR was not found to be a prognostic factor for medication response in most studies [23,33], and this negative finding was confirmed by a prospective study by Verrotti et al., who specifically addressed this issue [34]. Conversely, in another work PPR seemed associated with a lower rate of spontaneous remission without ASM during long-term follow-up in IGE patients [35]. 

### 3.3. EEG Slowing on Background Activity

When considering a manual assessment of the background activity, the presence of EEG slowing was found to be associated with a lack of medication response in CAE patients in two well-known studies in which baseline EEG was reviewed and patient outcome was assessed prospectively and/or retrospectively [36,37]. However, this finding was not replicated in the well-designed Dutch study, in which CAE diagnosis was made following strict diagnostic criteria, as mentioned previously [32]. Indeed, generalized background slowing should be considered an exclusion criterion for IGE diagnosis [3], limiting the significance of this EEG feature in CAE prognostic assessment. Background EEG slowing in CAE subjects needs to be carefully distinguished from the occurrence of occipital intermittent rhythmic delta activity (presenting as unilateral or bilateral runs of sinusoidal, regular, rhythmic, high-amplitude 2–3 Hz activity located over the posterior regions and enhanced by hyperventilation and drowsiness), which has been consistently associated with a favorable seizure outcome in different studies [38]. 

### 3.4. Other EEG Findings

Eye-closure sensitivity consists of GSWDs/GPSWDs that typically appear within 1–3 s after eye closure and last 1–4 s, which may occur across different IGE and non-IGE genetic generalized syndromes (typically in eyelid myoclonia with absences, which will not be discussed here) [39,40]. Among IGE syndromes, eye-closure sensitivity may be more commonly observed in JME patients, though it has also been described in absence epilepsies and in IGE with generalized tonic-clonic seizures alone [40]. A few authors examined the prognostic significance of eye-closure sensitivity in JME patients, with contrasting results, and solid evidence indicating a negative prognostic impact of this peculiar EEG feature has not been provided [41,42]. 

Following the study by Matsuoka et al. that described frequent activation of GSWDs/GPSWDs during demanding mental activities [15], a few authors explored the prognostic significance of ED and/or seizure induction by praxis, especially in JME patients. Even in this case, reported results were contrasting [43,44], and this topic should be better investigated in studies considering baseline EEGs. 

Finally, the occurrence of early cessation of interictal EDs after ASM initiation with respect to baseline EEG was found to be a reliable indicator of good seizure outcome in IGE in a study performed by Steinhoff et al. [45]. Several studies failed to demonstrate the prognostic significance of other EEG variables, such as the occurrence of GPSWDs, in various IGE syndromes [23,33,46,47,48].

## 4. EEG Biomarkers Found with paEEG Recordings Associated with Poor Seizure Outcome

### 4.1. EEG Findings Selectively Occurring during Sleep

The bidirectional relationship between sleep and epilepsy has long been investigated. While NREM sleep promotes the hypersynchrony of neuronal networks, thus facilitating the occurrence of EDs [49], poor seizure control can also disrupt sleep architecture and affect sleep quality [50]. Interest in using sleep EEG in order to better evaluate prognosis in patients with IGE first arose during the 1990s after the study by Michelucci and colleagues, who reported that generalized epilepsy patients with unfavorable long-term outcome and absences persisting in adult life exhibited polyspike runs during NREM sleep [51]. These polyspike runs were found to be similar to GPFA, the typical EEG pattern of Lennox-Gastaut syndrome [52]. GPFA was later described by another group in patients with atypical phenotypes who exhibited poor seizure control and borderline cognitive profile [53]. Since these first descriptions, GPFA has been considered a unique EEG pattern in patients showing intermediate phenotypes between idiopathic and symptomatic generalized epilepsies, but some authors have more recently described it in typical IGE syndromes as well, with contrasting results regarding their prognostic significance [11,54,55,56,57]. 

**Figure 3:**
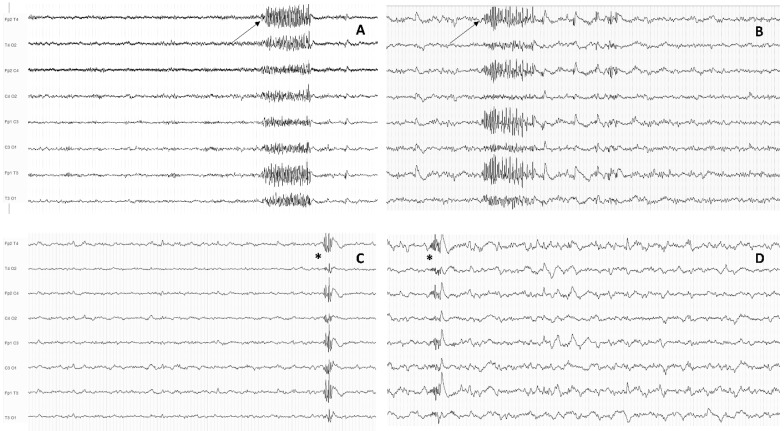
Examples of generalized paroxysmal fast activity (GPFA) and generalized polyspike train (GPT). Panel (**A**,**B**): examples of GPFA (black arrow); Panel (**C**,**D**): Examples of GPT (asterisk).

However, Sun et al. recently explored GPTs (defined as bursts of at least five spikes lasting less than 1 s) and GPFA (defined as bursts of at least 1 s within the beta frequency with a frontal predominance) occurring during sleep in a relatively large cohort of IGE patients (examples of GPFA and GPT are shown in Figure 3) [58]. 

All patients exhibiting GPFA also had GPTs, suggesting a shared pathophysiological mechanism between these two EEG features, with less impaired γ-aminobutyric-acid-mediated inhibition hypothesized in the latter. GPTs were significantly associated with drug resistance in multivariable analysis, a finding later corroborated in a replication cohort. Conversely, no significant association with prognosis was documented for the GPFA pattern, although this result might have been affected by the limited availability of sleep EEG in the discovery cohort (37 patients): in fact, GPFA was actually observed in 21.1% of drug-resistant patients and in no drug-responsive one. The prognostic significance of GPT and GPFA was later replicated in other single-center retrospective studies [59,60]. More recently, Kamitaki et al. developed a logistic regression model that discriminated ASM-responsive from ASM-refractory IGE patients with a fairly good accuracy [61]. The model included GPT and GSWD/GPSWD burden during sleep (along with some well-validated clinical variables), which were both found to be independently associated with ASM refractoriness. Similar findings were obtained by another recent work, in which both GPT/GPFA and a higher GSWD burden were associated with a lack of two-year remission at the last medical observation [62].

Unfortunately, all these studies were burdened by remarkable methodological limitations, particularly regarding their retrospective design and the timing of sleep EEG exams. Since only post-treatment paEEG recordings were evaluated in most patients, the predictive role of GPFA and GPT cannot be definitely confirmed, and these patterns might also represent an epiphenomenon of prolonged unremitting seizures in refractory patients. This hypothesis has been partially corroborated by the results of Jensen and colleagues, who tried to overcome the methodological limitations regarding the timing of EEG recording [63]. The authors investigated GPT in a cohort of IGE patients, for 79 of whom treatment-naïve EEG recordings were available. They found a very low (1.3%) prevalence of GPT and a low prognostic value of this pattern for subsequent treatment response. Conversely, when exploring GPT in EEGs of already treated patients with persistent seizures, a significant association with drug resistance at the last follow-up visit was found. In view of the discrepancy between treatment-naïve EEG and follow-up EEG, the authors speculated that the lack of ED suppression by ASM, rather than the specific interictal abnormalities, might represent an EEG marker of treatment resistance. However, the absence of 24-h EEG recordings in all patients may have determined an underestimation of GPT prevalence, considering that a previous study highlighted a median latency of 6.5 h to capture their first occurrence from the start of EEG recordings [59]. 

### 4.2. Generalized Discharge Duration Measured during 24-h EEG Recordings

Another EEG finding that has been investigated during the last decade as a potential biomarker of drug resistance is GSWD/GPSWD duration. Seneviratne et al. retrospectively reviewed the 24-h EEGs of 105 IGE patients and investigated the association between the duration of seizure remission (measured as the interval between the 24-h EEG recording and the last seizure) and several EEG features [64]. They analyzed the significance of atypical and focal EEG abnormalities, spike density (defined as the number of spikes per hour evaluated both during the 24-h period and separately during different physiological states), and the mean duration of generalized paroxysms. While no association between focal abnormalities and seizure freedom duration was found, the authors observed a statistically significant inverse association between longer periods of seizure remission and both longer EEG paroxysms and higher spike density. The retrospective assessment of seizure freedom did not help clarify the role of these EEG abnormalities in the prediction of seizure remission. Moreover, the study setting in a tertiary adult epilepsy center and the heterogeneity of the 24-h EEG recording delay from epilepsy diagnosis further limited the generalizability of this study’s findings. The prognostic significance of prolonged epileptiform EEG runs in the prediction of seizure remission was recently supported by the results of a study in JME patients, in which ED duration longer than 2.6 s correctly identified patients with seizure relapse within 12 months after paEEG [65].

However, in the aforementioned study conducted by Jensen and colleagues, [63] the prognostic significance of prolonged ED was not confirmed when specifically analyzing treatment naïve EEGs. Conversely, in post-treatment EEGs, ED duration was found to be associated with drug-resistance at the last visit. In conclusion, the specific role of prolonged GSWD/GPSWD runs in the prediction of seizure outcome in IGE should be better investigated in larger prospective studies.

## 5. The Emerging Role of Advanced EEG Analysis in the Identification of New Biomarkers of Medication Refractoriness

Neurophysiology practice is currently based mainly on the visual inspection of EEG recordings, which may provide only a limited amount of information, especially in the context of partially stereotyped EEG patterns, as in IGE [66]. In recent years, several computational techniques have been developed for the processing of EEG signals, which have allowed the investigation and analysis of subtle dynamics that are not detectable by visual inspection alone and may have a potential role as adjunctive prognostic indicators [67]. Advanced EEG analysis is especially important considering the great amount of information that could be extracted from spontaneous intrinsic brain activity in terms of both background activity and interictal/ictal epileptiform abnormalities. Indeed, an abnormal interaction between a hyperexcitable cortex and subcortical structures is thought to occur also outside ictal phases [68]. However, only a few studies have explored the prognostic role of computational analysis of EEG data in IGE, by using heterogeneous techniques ranging from conventional power analysis to emerging machine learning approaches.

The prognostic significance of EEG background slowing in IGE patients has been assessed through quantitative EEG power analysis by Abela and colleagues, who found a correlation between a shift to slower alpha rhythms, especially over posterior regions of the brain, and poor seizure control during the 12 months prior to study, suggesting a more pronounced cortical dysfunction in drug-resistant patients [69]. However, due to the enrollment of already treated patients, the impact of specific ASMs could not be properly ruled out. In addition, the use of retrospective outcome measures also limited the authors’ conclusions. The same topic was also addressed in other studies, with similar methodological limitations (i.e., inclusion of already treated patients with heterogenous ASMs, different timing of analyzed EEGs), which failed to confirm a significant association between background slowing and drug resistance [70].

Other authors explored EEG markers of treatment resistance by using current source density measures in ED-free resting-state EEGs to compare differences in pre and posttreatment EEG between responders and non-responders [71]. The authors analyzed average current source density to obtain a single value reflecting global electrical outward activity of the cortex. Based on previous evidence that global current source density values were increased in untreated IGE patients as compared to healthy subjects and that these values usually decreased after ASM treatment [72], they explored whether differences in these values after ASM treatment correlated with ASM response. They found that current source density decreases soon after treatment was strictly associated with treatment response, potentially helping in the early prognostic assessment of IGE patients with rare breakthrough seizures. 

In addition, Yeom et al. explored the prognostic significance of the localization of the GSWD generator in CAE patients [73], following previous functional magnetic resonance imaging studies which highlighted that cortical GSWD generators were associated with a less satisfactory seizure control compared with “classical” thalamic sources [74]. The researchers used exact low-resolution brain electromagnetic tomography analysis to examine current sources of averaged GSWDs and found a significantly higher involvement of the temporal lobe in drug-resistant CAE patients compared with ASM-responsive ones, in line with previous functional magnetic resonance findings.

Resting-state interictal background EEG (i.e., ED free) can be used to analyze functional connectivity between different cortical areas by measuring the statistical dependence between their signals. Several EEG-based functional connectivity measures have been developed based on amplitude, frequency, and phase [75,76]. Among these different measures, partial directed coherence is a well-validated technique that allows the intensity and directionality of functional connectivity between different brain areas to be inferred [77]. Recently, Canafoglia and colleagues investigated partial directed coherence in a cohort of CAE patients and demonstrated a more pronounced increase in the information outflow from the frontal regions to other brain regions in the alpha and beta band in non-responders to the first ASM compared with responders [78]. According to the authors, the frontal cortex might play a quantitatively stronger dysfunctional role in the generation of absences resistant to ASMs.

However, since IGE pathophysiology has been consistently attributed to the involvement of widespread brain regions and extensive networks, complex brain network investigation based on graph theory might be more valuable to identify markers of treatment resistance [79,80,81,82]. The strength of functional connectivity within and between brain networks may be represented as a graph of nodes (areas) connected by edges by “weighting” each edge according to the magnitude of the functional connectivity measure used [83]. This graph theoretical network approach has been recently used to demonstrate an association between interictal network topology in the alpha frequency band and seizure control in IGE patients [84]. In another well-designed study conducted on resting-state EEG in treatment-naïve JME patients, functional connectivity based on graph theoretical analysis was analyzed by means of coherence and phase locking value [85]. The authors found through both methods that global and local brain network efficiency was decreased in ASM-refractory JME patients compared with healthy controls, whereas such a difference was not found in ASM responders. The authors suggested that a more profound alteration in brain network topology might already be present at epilepsy onset in refractory JME. 

Additionally, microstate analysis is an increasingly applied method to assess EEG-based brain network dynamics in the millisecond range [86]. Past studies identified four reliable EEG microstates known as classes A, B, C, and D, which are respectively associated with fMRI resting-state activity within the sensorimotor, visual, salience, and attentional networks [87,88,89]. EEG microstate analysis may be particularly useful to assess resting-state brain network dynamics in IGE since temporal variability in network activity has been suggested to be a key feature in these patients [90]. Jiang et al. found that IGE patients who had experienced seizures in the previous two years showed different sensorimotor and salience network dynamics than seizure-free patients and healthy volunteers, as measured by EEG microstate [91]. 

Finally, other authors developed a new machine learning method to simultaneously assess several quantitative EEG features in drug-naïve IGE patients in order to automatically discriminate between responders and non-responders with artificial intelligence algorithms. Despite the low number of included patients, one study found an excellent discrimination ability of their support vector machine model based on 10 different EEG features [92]. Future studies should evaluate the predictive ability of the selected features in larger cohorts and investigate the ability of machine learning approaches to predict drug resistance in IGE patients considering also functional connectivity measures. 

## 6. Future Perspectives and Conclusions

After an extensive literature review, we could identify some EEG findings associated with treatment resistance in IGE patients, which are summarized in Table 1 (Table 1). The most promising EEG markers emerged from 24-h EEG studies and advanced EEG analysis. The 24-h EEG appears to be a useful tool to identify useful EEG markers for the clinical management of IGE patients due to its higher yield, ability to capture sleep quality and to measure GSWD duration. 

However, despite some interesting results, it is worth to mention that the previously highlighted limitations prevent us from recommending the use of these EEG patterns/features as prognostic biomarkers in routine clinical practice: in fact, prospective studies with systematic use of 24-h EEG recordings are warranted to draw more solid conclusions. Recently validated automated methods could be used to reduce interrater differences and the time needed to manually review paEEGs, thus allowing to conduct large-scale studies [93,94].

Regarding advanced EEG analysis, some of the reviewed computational techniques (including machine learning approaches) yielded excellent results and prompted future multicenter studies including large cohorts of patients to validate these findings. Apart from its prognostic impact, a better understanding of the quantitative EEG features and connectivity measures associated with treatment resistance may also provide crucial insights into the pathophysiological mechanisms underlying drug resistance in IGE, which may help the development of new treatments for refractory cases, including targeted neuromodulation approaches.

## Figures and Tables

**Table 1:** EEG markers associated with treatment resistance in idiopathic generalized epilepsy: a summary of current literature findings.

EEG Marker	Subjects	Methodology	Association with Treatment Resistance	Reference
Focal abnormalities	IGE, JME, JAE	Visual assessment of standard EEG—retrospective and prospective	Not associated	[16,17,18,19,20,21,22,23,24,25]
Photoparoxysmal response	IGE, CAE	Visual assessment of standard EEG—retrospective and prospective	Association in CAE, not useful in IGE in general	[23,26,27,28,29,33,34,35]
Eye closure sensitivity	JME	Visual assessment of standard EEG—retrospective	Not associated	[41,42]
Praxis-induction	JME	Visual assessment of standard EEG with neuropsychological activation—retrospective	Controversial	[43,44]
Generalized spike-wave and polyspike-wave discharge duration	IGE	24-h EEG recordings, sleep EEG recordings -retrospective	Yes, except in one study	[63,64,65]
Generalized paroxysmal fast activity and generalized polyspike train	IGE	24-h EEG recordings, sleep EEG recordings—retrospective	Yes, except in one study	[58,59,60,61,62,63]
Background slowing	IGE, CAE	Visual assessment of standard EEG, Quantitative analysis resting-state EEG—retrospective	Not associated, except in CAE	[36,37,69,70]
Frontal lobe connectivity	CAE	Partial directed coherence in resting state EEG—retrospective	Yes	[78]
Graph-theory based functional connectivity	JME	Coherence and phase locking value on resting state EEG—retrospective	Yes	[85]
Microstates analysis	IGE	Microstate analysis on resting-state EEG—retrospective	Yes	[91]
Machine-learning on quantitative EEG features	IGE	Support-vector-machine model on resting-state EEG quantitative features—retrospective	Yes	[92]

Abbreviations: CAE = childhood absence epilepsy; EEG = electroencephalography; GPSWD = generalized polyspike-wave discharge; GSWD = generalized spike wave discharge; IGE = idiopathic generalized epilepsy; JAE = juvenile absence epilepsy; JME = juvenile myoclonic epilepsy.

## Data Availability

Not applicable.

## Abbreviations

ASM = antiseizure medication; CAE = childhood absence epilepsy; ED = epileptiform discharge; EEG = electroencephalography; GPFA = generalized paroxysmal fast activity; GPSWD = generalized polyspike-wave discharge; GPT = generalized polyspike train; GSWD = generalized spike-wave discharge; IGE = idiopathic generalized epilepsy; JME = juvenile myoclonic epilepsy; NREM = non-rapid eye movement; paEEG = prolonged ambulatory EEG.
